# Elevated serum Raftlin levels in non-muscle invasive bladder cancer: diagnostic implications and rs690037 genotype analysis

**DOI:** 10.1590/1806-9282.20251628

**Published:** 2026-06-15

**Authors:** Senay Balci, Rojda Tanriverdi, Ali Nebioğlu, Didem Derici Yildirim, Cemil Gülüm, Murat Bozlu, Lülüfer Tamer

**Affiliations:** 1 Mersin University, Faculty of Medicine, Department of Biochemistry – Mersin, Turkey.; 2 Mersin City Training and Research Hospital, Department of Urology – Mersin, Turkey.; 3 Mersin University, Faculty of Medicine, Department of Biostatistics and Medical Informatics – Mersin, Turkey.; 4 Mersin University, Department of Chemistry – Mersin, Turkey.; 5 Mersin University, Department of Urology – Mersin, Turkey.

**Keywords:** Raftlin, Non-muscle invasive bladder cancer, Biomarker, RFTN1, Polymorphism

## Abstract

**OBJECTIVE:**

Non-muscle invasive bladder cancer is a common urological malignancy characterized by high recurrence and progression rates. There is a critical need for non-invasive biomarkers to support early detection and disease monitoring. Raftlin, a lipid raft-associated scaffold protein involved in immune regulation and signal transduction, has recently gained attention in cancer biology. The aim of this study was to investigate serum Raftlin levels and the rs690037 polymorphism of the Raftlin gene in non-muscle invasive bladder cancer patients.

**METHODS:**

A case-control study was conducted, including 30 patients diagnosed with non-muscle invasive bladder cancer and 50 healthy controls. Serum Raftlin levels were measured using enzyme-linked immunosorbent assay, and the rs690037 polymorphism was analyzed using real-time polymerase chain reaction. Gender-stratified analysis and receiver operating characteristic curve analysis were performed. Clinical and pathological characteristics including tumor stage, grade, and European Organisation for Research and Treatment of Cancer risk groups, were analyzed.

**RESULTS:**

Serum Raftlin levels were significantly elevated in non-muscle invasive bladder cancer patients compared to healthy controls (p<0.001). Gender-stratified analysis confirmed this association was independent of gender (males: p<0.001; females: p<0.001). Receiver operating characteristic curve analysis demonstrated an area under the curve of 0.987 (95%CI 0.966–1.000), with an optimal cut-off of 5.28 ng/mL providing 96.7% sensitivity and 98.0% specificity. Trends toward higher Raftlin levels were observed in more aggressive disease features. No significant association was found between the rs690037 polymorphism and disease presence.

**CONCLUSION:**

This pilot study suggests that serum Raftlin levels are significantly elevated in non-muscle invasive bladder cancer and show high discriminatory ability. While preliminary findings suggest potential as a non-invasive biomarker, validation in larger, independent cohorts is required before clinical implementation.

## INTRODUCTION

Bladder cancer (BC) is one of the most common malignancies worldwide, with an incidence approximately three times higher in males than in females^1,2^. Non-muscle invasive bladder cancer (NMIBC), encompassing tumors limited to the mucosa or lamina propria, represents about 75% of all BC cases^3^. NMIBC poses substantial clinical challenges due to its high recurrence rate (50–70%) and risk of progression to muscle-invasive disease (10–30%)^2,4,5^. Therefore, there is an urgent need to develop non-invasive biomarkers for early diagnosis and prediction of disease progression^4^.

In recent years, protein-based biomarkers have gained considerable attention in the diagnosis and monitoring of BC^6^. Among these, lipid raft-associated proteins have emerged as promising candidates. Lipid rafts are specialized membrane microdomains that facilitate cell signaling, receptor clustering, and intercellular communication. These microdomains are linked to several oncogenic pathways such as Insulin-like Growth Factor, Phosphoinositide 3-kinase/Protein kinase B, Vascular Endothelial Growth Factor/Vascular Endothelial Growth Factor Receptor 2, and Cluster of Differentiation 44, and are known to regulate tumor proliferation, migration, and drug resistance^7,8^.

Additionally, lipid rafts represent critical regulatory platforms in the context of cancer stem cells (CSCs) within the tumor microenvironment. Evidence shows that CSCs exhibit significantly higher lipid raft levels compared to other cancer cells, and CSC markers such as CD24, CD44, and CD133 are localized to these microdomains. Lipid rafts facilitate CSC self-renewal, epithelial–mesenchymal transition (EMT), drug resistance, and niche maintenance^9^.

Raftlin (RFTN1), a scaffold protein for lipid rafts, plays a key role in B-cell receptor (BCR) signaling^8^. Recent insights suggest that RFTN1 may also modulate inflammatory mechanisms mediated by Toll-like receptor (TLR) pathways and immune cell differentiation. Specifically, RFTN1 interacts with nuclear receptor Retinoic acid receptor-related orphan receptor gamma t (RORγt) to promote interleukin 17 (IL-17) expression and enhance pathogenic Th17 differentiation, linking it to pro-inflammatory processes^9^. By supporting lipid raft integrity, RFTN1 may influence inflammation-driven tumor microenvironments.

Emerging studies also implicate RFTN1 in tumor cell proliferation, migration, and cellular adhesion. For instance, RFTN1 overexpression has been associated with increased proliferation in non-small cell lung cancer (NSCLC)^10^, and involvement in adhesion-related signaling in various cancers^11^.

Although direct data on the RFTN1 rs690037 polymorphism are limited, genetic variants such as this may affect protein expression or function, potentially influencing cancer susceptibility. The rs690037 variant has been previously associated with primary open-angle glaucoma^12^, suggesting potential functional consequences that warrant investigation in other disease contexts.

This study aimed to evaluate serum RFTN1 levels in NMIBC patients and investigate the association of the rs690037 polymorphism with disease risk. Moreover, we analyzed the relationship between these parameters and clinical/pathological features to assess RFTN1's potential as a biomarker in NMIBC.

## METHODS

### Study population

Between February 2024 and June 2024, data from 30 patients who were admitted to the Urology Clinic of Mersin University and followed and treated with a diagnosis of NMIBC, as well as 50 healthy individuals with no history of malignancy, were prospectively analyzed. Participants with invasive and/or metastatic BC, those under the age of 18, patients with malignancies other than BC, and individuals who did not provide informed consent were excluded from the study.

Sociodemographic data, including age, weight, height, body mass index (BMI), family history, and smoking status, were collected for all participants included in the study. Written informed consent forms were obtained from all individuals participating in the study.

Specific ethical approval, numbered 2024/089 and dated January 31, 2024, was obtained from the Clinical Research Ethics Committee (Institutional Review Board) of Mersin University Faculty of Medicine, focusing on RFTN1 analysis in bladder cancer, in accordance with the Helsinki Declaration for the current study.

### Sample collection and storage

The analysis of serum RFTN1 levels and the detection of the RFTN1 gene polymorphism rs690037 were performed at the Medical Biochemistry Laboratory of Mersin University. For polymorphism analysis, blood samples were collected from both patients and healthy individuals into 2 mL ethylenediaminetetraacetic acid (EDTA) tubes. For the enzyme-linked immunosorbent assay (ELISA), blood samples were drawn into 5 mL plain biochemistry tubes.

The blood samples in the plain biochemistry tubes were centrifuged at 4,000 rpm for 10 min, and the serum was separated. Storage of serum samples at -80°C retarded protein degradation, thereby preserving the long-term stability of biomolecules such as RFTN1 and enhancing the reliability of the research results. Therefore, serum samples were maintained at -80°C until the day of analysis. Blood samples collected in EDTA tubes were stored at +4°C.

### Measurement of Raftlin levels

Serum RFTN1 levels were measured using a commercial BT Lab ELISA kit (E4717Hu). Assay precision was evaluated by repeated measurements of random samples within the same batch, and intra-assay precision was assessed by analyzing three serum samples (n=18 each), yielding coefficients of variation (CV%) of 6.1, 5.4, and 5.9%, respectively.

The assay sensitivity was 0.41 ng/mL, with a detection range of 0.5–40 ng/mL. Standard curves were generated using recombinant RFTN1 standards at concentrations of 0, 1.5, 3, 6, 12, and 24 ng/mL, in accordance with the manufacturer's protocol.

This process ensures that the quantification of RFTN1 is both accurate and reliable, as it is based on comparison with established standards and validated analytical performance. The utilization of a four-parameter logistic model represents the standard approach in ELISA assays to translate optical density readings into precise concentration values across the full dynamic range of the assay.

### DNA isolation and polymorphism analysis

DNA isolation from blood samples stored at +4°C was performed using a commercial DNA isolation kit (Roche Diagnostics, Mannheim, Germany). The isolated DNA samples were then analyzed for the RFTN1 gene polymorphism rs690037 using a real-time polymerase chain reaction (PCR) device (Roche LightCycler 480; Roche Diagnostics, Mannheim, Germany).

This approach ensures high-quality DNA extraction and precise detection of genetic polymorphisms. Real-time PCR represents a sensitive and specific method widely employed for genotyping, enabling accurate identification of the rs690037 polymorphism in the RFTN1 gene.

### Statistical analysis

Statistical analyses of the data obtained in this study were performed using the trial version of the Statistical Package for the Social Sciences (SPSS) 25.0 software package (SPSS, IBM Corp., Armonk, NY, USA). The distribution of continuous variables was assessed with the Kolmogorov-Smirnov test. For data that did not show a normal distribution, descriptive statistics were presented as median and interquartile range (25th and 75th percentiles). Categorical variables were expressed as numbers and percentages (%).

To compare gender and genotype distributions between groups, the chi-square test was applied. For continuous variables such as age and serum RFTN1 levels, the non-parametric Mann-Whitney U test was used. Gender-stratified analysis was performed to evaluate RFTN1 levels separately in male and female subgroups. Hardy-Weinberg equilibrium analysis for genetic data was evaluated separately for patient and control groups using the chi-square test.

Receiver operating characteristic (ROC) curve analysis was performed to assess the diagnostic performance of serum RFTN1 levels in discriminating NMIBC patients from healthy controls. The area under the curve (AUC), optimal cut-off value, sensitivity, and specificity were calculated. The optimal cut-off point was determined using the Youden Index (sensitivity+specificity-1). In all statistical analyses, a p-value of less than 0.05 was considered statistically significant.

## RESULTS

A total of 80 individuals were included in the study. Of these, 30 comprised the patient group diagnosed with NMIBC, while 50 constituted the healthy control group. The distribution of participants’ age, gender, sociodemographic, and clinical characteristics is summarized in Table 1. There was no statistically significant difference in age between the patient and control groups (p=0.313). However, the proportion of males was found to be significantly higher in the patient group compared to the control group (p=0.001).

**Table 1:** Sociodemographic and clinical characteristics of the study groups.

Variable		Control group (n=50)	NMIBC group (n=30)	Total (n=80)	p-value
**Gender, n (%)**					
	Female	31 (62.0%)	7 (23.3%)	38 (47.5%)	**0.001**
	Male	19 (38.0%)	23 (76.7%)	42 (52.5%)	
Age, mean±SD (years)		65.44±5.54	66.87±6.92	–	0.313
Comorbid disease, n (%)		20 (40.0%)	12 (40.0%)	32 (40.0%)	0.999
BMI, mean±SD (kg/m²)		26.2±2.1	26.1±2.0	–	0.872
Family history, n (%)		15 (30.0%)	9 (30.0%)	24 (30.0%)	0.999
Smoking, n (%)		25 (50.0%)	15 (50.0%)	40 (50.0%)	0.999

NMIBC: non-muscle invasive bladder cancer; BMI: body mass index; SD: standard deviation. Bold values indicate statistically significant differences (p<0.05).

Regarding genotype distributions, no statistically significant difference was observed between the groups (p=0.439). In addition, Hardy-Weinberg equilibrium analysis revealed no significant deviation in either group (p=0.284 for the patient group, p=0.428 for the control group) (Table 2).

**Table 2:** Distribution of RS690037 genotypes in patient and control groups.

Genotype	Control group n (%)	NMIBC group n (%)	Total n (%)	p-value
TT	12 (24.0%)	5 (16.7%)	17 (21.3%)	0.439
TC	21 (42.0%)	17 (56.7%)	38 (47.5%)	
CC	17 (34.0%)	8 (26.7%)	25 (31.3%)	

NMIBC: non-muscle invasive bladder cancer; TT: homozygous T allele; TC: heterozygous (T/C); CC: homozygous C allele.

Serum RFTN1 levels did not exhibit a normal distribution in either the patient or control groups (p<0.001). Therefore, descriptive statistics are presented as median and 25th–75th percentiles. Serum RFTN1 levels were found to be statistically significantly elevated in the NMIBC group compared to the control group (p<0.001). The distribution of serum RFTN1 levels according to groups is presented in Table 3.

**Table 3:** Serum Raftlin levels and tests of normality in patient and control groups.

Group	n	Median (ng/mL)	25th percentile	75th percentile	Minimum	Maximum	p-value
Control	50	3.20	2.47	3.59	1.26	4.52	<0.001
NMIBC	30	7.62	7.05	8.61	5.04	12.20	

NMIBC: non-muscle invasive bladder cancer. Note: Both Kolmogorov-Smirnov and Shapiro-Wilk tests indicate that serum RFTN1 levels do not follow a normal distribution (p<0.05). Therefore, serum RFTN1 levels are presented as median [25th–75th percentile]. The Mann-Whitney U test was used for comparison.

Given the significant gender imbalance between groups (76.7% male in NMIBC vs. 38.0% in controls), a gender-stratified analysis was performed. In male subjects, serum RFTN1 levels were significantly higher in NMIBC patients (median: 7.48 ng/mL, interquartile range [IQR]: 6.92–8.45) compared to male controls (median: 3.15 ng/mL, IQR: 2.40–3.52; p<0.001). Similarly, in female subjects, RFTN1 levels were significantly elevated in NMIBC patients (median: 8.12 ng/mL, IQR: 7.35–9.20) compared to female controls (median: 3.24 ng/mL, IQR: 2.51–3.64; p<0.001). These results are summarized in Table 4, demonstrating that the association between RFTN1 and NMIBC is independent of gender.

**Table 4:** Gender-stratified analysis of serum Raftlin levels.

Gender	Group	N	Median (ng/mL)	25th percentile	75th percentile	p-value
Male	Control	19	3.15	2.40	3.52	<0.001
	NMIBC	23	7.48	6.92	8.45	
Female	Control	31	3.24	2.51	3.64	<0.001
	NMIBC	7	8.12	7.35	9.20	

NMIBC: non-muscle invasive bladder cancer. Note: The Mann-Whitney U test was used for comparison within each gender subgroup.

Within the NMIBC patient group (n=30), clinical and pathological data were available and analyzed. The distribution included 18 patients (60.0%) with Ta stage tumors, 10 patients (33.3%) with T1 stage, and 2 patients (6.7%) with carcinoma in situ (CIS). Regarding tumor grade, 11 patients (36.7%) had low-grade tumors and 19 patients (63.3%) had high-grade tumors. According to European Organisation for Research and Treatment of Cancer (EORTC) risk stratification, 9 patients (30.0%) were classified as low risk, 12 patients (40.0%) as intermediate risk, and 9 patients (30.0%) as high risk.

Serum RFTN1 levels showed a trend toward higher values in patients with more aggressive disease features, though statistical significance was limited by sample size. RFTN1 levels were numerically higher in T1 tumors (median: 8.05 ng/mL) compared to Ta tumors (median: 7.38 ng/mL), and in high-grade tumors (median: 7.89 ng/mL) compared to low-grade tumors (median: 7.21 ng/mL). When stratified by EORTC risk groups, RFTN1 levels showed progressive elevation from low-risk (median: 7.15 ng/mL) to intermediate-risk (median: 7.62 ng/mL) to high-risk patients (median: 8.28 ng/mL). These findings are presented in Table 5.

**Table 5:** Clinical and pathological characteristics of non-muscle invasive bladder cancer patients and association with serum Raftlin levels.

Characteristic		n (%)	Median RFTN1 (ng/mL)	25th percentile	75th percentile
Tumor stage					
	Ta	18 (60.0%)	7.38	6.85	8.42
	T1	10 (33.3%)	8.05	7.28	8.95
	CIS	2 (6.7%)	7.82	7.15	8.49
Tumor grade					
	Low-grade	11 (36.7%)	7.21	6.78	7.95
	High-grade	19 (63.3%)	7.89	7.18	8.82
EORTC risk group					
	Low risk	9 (30.0%)	7.15	6.68	7.88
	Intermediate risk	12 (40.0%)	7.62	7.05	8.45
	High risk	9 (30.0%)	8.28	7.52	9.28

CIS: carcinoma in situ; EORTC: European Organisation for Research and Treatment of Cancer; RFTN1: Raftlin.

**Figure 1:**
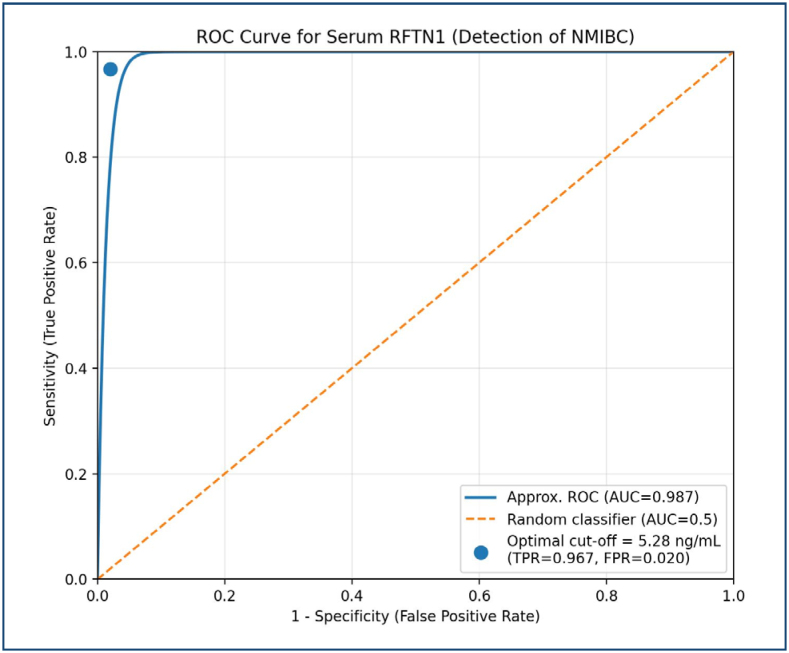
Diagnostic performance of serum Raftlin for detection of non-muscle invasive bladder cancer. AUC: area under the curve. Note: Optimal cut-off value was determined using the Youden Index.

ROC curve analysis was performed to evaluate the diagnostic performance of serum RFTN1 in discriminating NMIBC patients from healthy controls. The analysis yielded an AUC of 0.987 (95%CI 0.966–1.000; p<0.001), indicating a high level of discriminatory performance within this study population. Using the Youden Index, the optimal cut-off value was determined to be 5.28 ng/mL, which provided a sensitivity of 96.7% (95%CI 82.8–99.9%) and a specificity of 98.0% (95%CI 89.4–99.9%) for detecting NMIBC. The positive predictive value was 96.7%, and the negative predictive value was 98.0%. These results are summarized in Figure 1.

In summary, serum RFTN1 levels were found to be significantly elevated in the NMIBC patient group across both genders. The gender-stratified analysis confirmed that elevated RFTN1 is independently associated with NMIBC. Clinical correlation analysis revealed trends toward higher RFTN1 levels in more aggressive disease subtypes. The ROC curve analysis demonstrated excellent diagnostic performance, supporting the potential utility of serum RFTN1 as a biomarker for NMIBC detection.

## DISCUSSION

In this study, the relationship between serum RFTN1 levels and the rs690037 polymorphism of the RFTN1 gene was evaluated in patients diagnosed with NMIBC. The results demonstrated a statistically significant elevation in serum RFTN1 levels in the NMIBC group compared to healthy controls, while no significant differences were observed in the distribution of rs690037 genotypes. These findings highlight the potential diagnostic relevance of RFTN1 as a non-invasive biomarker in BC and warrant further mechanistic investigations.

RFTN1 is a scaffold protein localized within lipid rafts—specialized microdomains of the plasma membrane involved in protein clustering, receptor organization, and signal transduction. Originally characterized for its role in BCR signaling^8^, RFTN1 has more recently emerged as a modulator of signaling cascades relevant to cancer biology. Elevated RFTN1 expression has been identified in several solid tumors, including NSCLC^10^ and other malignancies^11^. The increased RFTN1 levels observed in NMIBC patients may reflect tumor-driven upregulation or active release into the bloodstream.

RFTN1 has been implicated in immunomodulatory pathways. Recent evidence indicates that RFTN1 interacts with nuclear receptor RORγt to promote IL-17 expression and enhance pathogenic Th17 differentiation^9^, linking it to pro-inflammatory processes in the tumor microenvironment^13^. RFTN1 has also been implicated in angiogenesis, where it is recruited to the VEGFR2 complex via neuropilin-1 (NRP1), facilitating VEGF-mediated angiogenic signaling^14^ a mechanism relevant to tumor growth and vascular remodeling.

The gender-stratified analysis in our study demonstrated that elevated RFTN1 levels were significantly associated with NMIBC in both male and female subgroups, confirming that this association is independent of the observed gender imbalance between groups. Furthermore, the ROC curve analysis yielded an AUC of 0.987, indicating a high level of discriminatory performance for distinguishing NMIBC patients from controls in the present study. The optimal cut-off of 5.28 ng/mL provided sensitivity of 96.7% and specificity of 98.0%, supporting the potential diagnostic utility of serum RFTN1.

Clinical correlation analysis revealed trends toward higher RFTN1 levels in patients with more aggressive disease features, including T1 stage, high-grade tumors, and high EORTC risk classification. However, statistical significance was limited by the modest sample size, and these observations require validation in larger cohorts.

The absence of a statistically significant difference in rs690037 genotype distributions between patients and controls suggests that the observed increase in serum RFTN1 levels may not be primarily driven by this specific genetic variant. RFTN1 regulation in NMIBC is more likely mediated through epigenetic modifications, post-translational alterations, or tumor microenvironmental stimuli. The rs690037 variant, although previously associated with primary open-angle glaucoma^12^, may lack functional consequences in the context of BC, or the sample size may have been insufficient to detect small genetic effects.

Our findings support the hypothesis that RFTN1 is involved in the pathophysiology of NMIBC. The clinical utility of RFTN1 as a biomarker may be enhanced by integrating it into a panel with other novel markers such as RNF19A^15^. Future studies should evaluate RFTN1 levels alongside tumor stage, grade, recurrence risk, and treatment response in larger, prospective cohorts.

## CONCLUSION

This pilot study suggests that serum RFTN1 levels are significantly elevated in patients with NMIBC compared to healthy controls, and this association remained significant after gender-stratified analysis. ROC curve analysis indicated a high level of discriminatory performance (AUC: 0.987), with high sensitivity and specificity in the present study. Clinical correlation analysis revealed trends toward higher RFTN1 levels in more aggressive disease features; however, these observations were limited by the small sample size. The rs690037 polymorphism showed no significant association with disease susceptibility, suggesting that RFTN1 regulation in NMIBC may be influenced predominantly by non-genetic mechanisms.

Despite the promising preliminary findings, several important limitations must be acknowledged. The modest sample size (n=30 patients) limits statistical power, particularly for subgroup analyses and genetic associations. The gender imbalance between groups, while addressed through stratified analysis, highlights the need for matched control groups in future studies. Additionally, the cross-sectional design precludes assessment of RFTN1's utility in monitoring disease recurrence or progression.

Future validation studies in larger, independent cohorts are essential to confirm these findings and establish RFTN1's clinical utility. Prospective studies should evaluate RFTN1 levels in relation to disease recurrence, progression, and treatment response. Integration of RFTN1 into multi-marker panels, potentially including established biomarkers and emerging candidates such as RNF19A^15^, may enhance diagnostic and prognostic accuracy. Functional studies are also warranted to elucidate the mechanistic role of RFTN1 in NMIBC pathophysiology. Only after such comprehensive validation can RFTN1 be considered for clinical implementation as a biomarker in NMIBC management.

## ETHICAL APPROVAL

Approved by the Clinical Research Ethics Committee of Mersin University Faculty of Medicine (Decision date: 31.01.2024; Decision number: 2024/089).

## Data Availability

The datasets generated and/or analyzed during the current study are available from the corresponding author upon reasonable request.
